# *Salmonella*–Host Interactions – Modulation of the Host Innate Immune System

**DOI:** 10.3389/fimmu.2014.00481

**Published:** 2014-10-07

**Authors:** Daniel Hurley, Matthew P. McCusker, Séamus Fanning, Marta Martins

**Affiliations:** ^1^School of Public Health, Physiotherapy and Population Science, UCD Centre for Food Safety, UCD Centre for Molecular Innovation and Drug Discovery, University College Dublin, Dublin, Ireland

**Keywords:** gastroenteritis, host innate immunity, macrophages, NTS, pathogenicity islands, salmonellosis

## Abstract

*Salmonella enterica* (*S. enterica*) are Gram-negative bacteria that can invade a broad range of hosts causing both acute and chronic infections. This phenotype is related to its ability to replicate and persist within non-phagocytic host epithelial cells as well as phagocytic dendritic cells and macrophages of the innate immune system. Infection with *S. enterica* manifests itself through a broad range of clinical symptoms and can result in asymptomatic carriage, gastroenteritis, systemic disease such as typhoid fever and in severe cases, death (1). Exposure to *S. enterica* serovars Typhi and Paratyphi exhibits clinical symptoms including diarrhea, fatigue, fever, and temperature fluctuations. Other serovars such as the non-typhoidal *Salmonella* (NTS), of which there are over 2,500, are commonly contracted as, but not limited to, food-borne sources causing gastrointestinal symptoms, which include diarrhea and vomiting. The availability of complete genome sequences for many *S. enterica* serovars has facilitated research into the genetic determinants of virulence for this pathogen. This work has led to the identification of important bacterial components, including flagella, type III secretion systems, lipopolysaccharides, and *Salmonella* pathogenicity islands, all of which support the intracellular life cycle of *S. enterica*. Studies focusing on the host–pathogen interaction have provided insights into receptor activation of the innate immune system. Therefore, characterizing the host–*S. enterica* interaction is critical to understand the pathogenicity of the bacteria in a clinically relevant context. This review outlines salmonellosis and the clinical manifestations between typhoidal and NTS infections as well as discussing the host immune response to infection and the models that are being used to elucidate the mechanisms involved in *Salmonella* pathogenicity.

## Introduction

Every year, thousands of cases of salmonellosis are reported worldwide. However, the actual number of infections may be very different and many times greater than expected since many milder cases are not diagnosed or reported (http://www.cdc.gov/salmonella). *Salmonella* infection or the disease associated with it, salmonellosis, is most often characterized by enteritis. However, host restricted serotypes tend to induce higher levels of bacteremia, while some human restricted serotypes cause a systemic disease that is characterized by mild symptoms (2). Children are the most likely group of individuals to present salmonellosis. The rate of diagnosed infections in children <5 years old is higher than the rate diagnosed in all other persons. Other groups of risk, such as the elderly and immunocompromised individuals are the most likely to present severe forms of the disease.

Persons with diarrhea usually recover completely after a few days of the initial infection, although it may be several months before their bowel habits return to normal. Contrary to this could be a small number of persons with *Salmonella* infections that develop pain in their joints, irritation of the eyes, and painful urination. Taken together, these symptoms indicate a disease called reactive arthritis. This disease can last for months or years, and can lead to chronic arthritis, which is extremely difficult to treat. Antibiotic treatment does not make a difference in whether or not the person develops arthritis (3). Other types of invasive infections caused by *Salmonella*, such as bacteremia, osteomyelitis, and meningitis, may also occur and in these cases may require antimicrobial therapy (4).

The continuous evolution of *Salmonella* at the genetic and genomic levels contributes to the increased virulence and resistance to multiple antibiotics, leading to a phenotype of multidrug resistance. This resistance is a significant public health concern (5). Two major changes in the epidemiology of non-typhoidal salmonellosis have occurred in the last century. These were the emergence of food-borne human infections caused by *Salmonella enterica* Enteriditis and by multidrug-resistant strains of *Salmonella enterica* Typhimurium. In this century, a concerning situation is the increased resistance that non-typhoidal *Salmonella* (NTS) presents to fluoroquinolones and third-generation cephalosporins. Clinical isolates showing carbapenem resistance have also being reported (4). In terms of therapy, treatment with antibiotics is not usually recommended for uncomplicated *Salmonella* gastroenteritis. However, recent studies indicated that a 3–5 days therapy with ceftriaxone for patients with severe gastroenteritis could lead to a faster recovery. A continuous surveillance scheme of *Salmonella* infections in both humans and animals is of importance. A better understanding of the mechanisms that can lead to the emergence of antimicrobial resistance in *Salmonella* may help develop better interventional strategies that can ultimately reduce the spread of resistant *Salmonella* between humans and reservoirs identified (or not) along the food chain.

Due to the importance of *Salmonella* in the clinical and public health setting, there has been a significant effort to deepen the knowledge about pathogenic determinants of this bacterium. The clinical relevance of the disease, associated with the advances on the molecular tools available to study *Salmonella* and the development of suitable animal models, have lead to the development of optimal conditions to drive the scientific community to generate a large expansion of our knowledge about the pathogenesis of *Salmonella*-induced enterocolitis (6). This research effort has also generated an increased amount of information on the host immune mechanisms that complements gaps that still exist in fundamental research developed in this area.

The goal of this review is to discuss salmonellosis, the clinical signs caused by *Salmonella* infections, and the advances in our knowledge on the innate intestinal immunity. Additionally, the interaction with the host and the models used to elucidate the mechanisms triggered by the interaction of *Salmonella* with the host will also be discussed.

## Interactions of *Salmonella* with the Gut Microbiome

The intestinal microbiome, which is host to an estimated 1 × 10^14^ bacteria, is responsible for conferring numerous aspects of the host response against salmonellosis (7). As many as 1,000 species of bacteria inhabit this niche, with the majority being classified as Gram-positive Actinobacteria and Firmicutes as well as Gram-negative bacteroides (8). A healthy gut microbiome provides protection against epithelial cell invasion via a series of strategies including the production of toxic metabolites, which have been shown to repress the expression of *Salmonella* virulence genes among others. This feature assists in the clearance of pathogens from the gut lumen after NTS-induced diarrhea (7). Increased fecal shedding and establishment of carrier status is commonly associated with prolonged treatment with antimicrobial compounds as these can have adverse effects on the composition of the gut microbiome of an individual (8, 9). This depletion of the natural gut microbiome may have long lasting effects and can result in an increased susceptibility to *Salmonella* colonization. One such example of this scenario is *S*. Typhimurium, which takes advantage of the availability of ethanolamine, a nutrient present in the microbiome, to gain a significant growth advantage in the intestine during inflammation over potential competing pathogens. *S*. Typhimurium-encoded virulence factors have been shown to induce the production of an alternate electron acceptor by the host, which supports anaerobic respiration and enables *S*. Typhimurium to outcompete other fermenting gut microbes sharing the same ecological niche (10).

## Salmonellosis

Salmonellosis causes significant morbidity and mortality on a global scale and occurs after the ingestion of food or water sources that have been previously contaminated by the fecal or urinary excretions of animals that can act as reservoirs of *Salmonella* (11). Following infection with *Salmonella* species, a broad range of clinical manifestations can be presented in a number of ways depending on the susceptibility of the host (12, 13). These include bacteremia, enteric fever, enterocolitis, and chronic asymptomatic carriage. Typhoid and Paratyphoid fever, collectively termed enteric fever, are contracted following infection with *S. enterica* serovars Typhi (*S*. Typhi) and Paratyphi (*S*. Paratyphi), respectively. In contrast, gastroenteritis is commonly associated with NTS serovars such as Typhimurium (*S*. Typhimurium) and Enteritidis (*S*. Enteritidis).

In human beings, *S*. Typhi and *S*. Paratyphi cause typhoid fever, a bacteremic illness, which presents in a unique manner when compared with other Gram-negative bacteremias (14, 15). *S*. Typhi has previously adapted to infect human hosts whereas other serovars have retained a broad host preference and are capable of infecting a range of animals causing enterocolitis (16). Serovars of *S. enterica* including Choleraesuis (*S*. Choleraesuis), Dublin (*S*. Dublin), and *S*. Typhimurium can successfully infect both human and animal hosts. However, the infection presents differently in each. Human infection with *S*. Choleraesuis and *S*. Dublin commonly results in bacteremia. In mice, *S*. Typhimurium causes symptoms similar to human typhoid fever and will disseminate throughout the body of the host causing systemic illness (17, 18). Systemic infection can result in a diverse range of clinical manifestations that include bradycardia, hepatomegaly, and splenomegaly. Bacterial emboli form skin lesions known as Rose spots that occur in approximately 30% of typhoid fever cases. NTS serovars cause self-limiting diarrhea and in rare cases, secondary bacteremia. Primary NTS bacteremia has also been reported in immunocompromised hosts (19, 20). Death from salmonellosis can be caused by perforation of the gut and necrosis of Peyer’s patches leading to peritonitis or toxic encephalopathy [H. (15)].

## *Salmonella* Species and Subspecies

**Figure 1:**
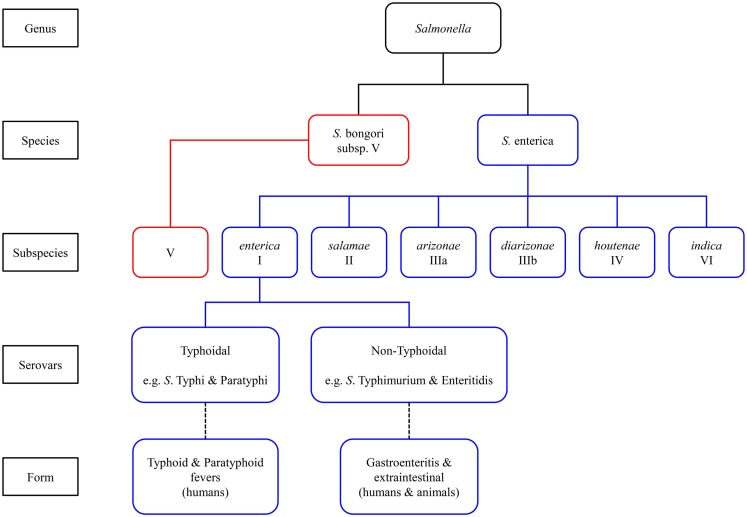
**Classification of *Salmonella* species and subspecies**.

*Salmonella enterica* are Gram-negative facultative intracellular anaerobes that can invade a broad range of hosts causing both acute and chronic infections by means of their ability to replicate and persist within non-phagocytic epithelial cells as well as phagocytic dendritic cells and macrophages of the host innate immune system (21, 22). The genus *Salmonella* comprises two species, *S. enterica* and *S. bongori* (also referred to as *subsp*. V). The former is further divided into six subspecies (as shown in Figure 1), which are biochemically differentiated into serovars based on the composition of their carbohydrate, flagellar, and lipopolysaccharide (LPS) structures. All *Salmonella* serotypes can be designated by an antigenic formula based on somatic (O) and flagellar (H) antigens in addition to capsular (Vi) antigens (16).

## *Salmonella* Pathogenicity Islands

Using *ex vivo* and *in vivo* animal models of infection, many virulence factors have been determined, which are responsible for inducing an inflammatory immune response in the infected host. There are two broad categories of proinflammatory stimuli that can be observed during *Salmonella* infection. These are pathogen-associated factors that stimulate the innate immune system of the host and virulence associated factors that exploit host processes resulting in disease pathology.

**Figure 2:**
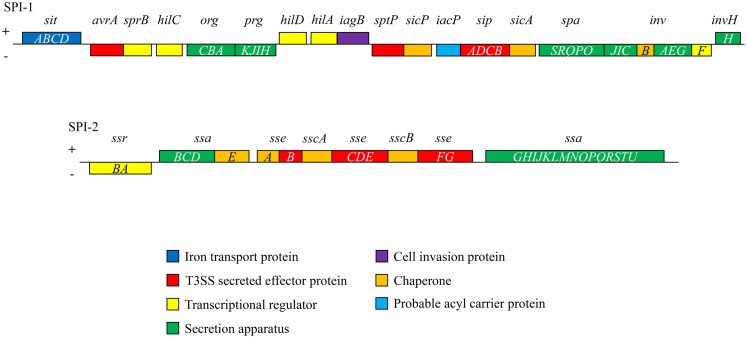
**Schematic illustration of the genes of SPI-1 and SPI-2 indicating their functional categories is shown**. In *Salmonella*, SPI-1 and SPI-2 encode a range of effector proteins, secretion apparatus, and transcriptional regulators in addition to T3SS-1 and T3SS-2.

*Salmonella* pathogenicity islands (SPI), historically acquired through horizontal gene transfer events, include clusters of genes, which encode the mechanisms through which *Salmonella* acts as a virulent pathogen (23, 24). These genetic islands are located on the bacterial chromosome or on plasmids, however, not all serovars possess every known SPI. SPI-1 through SPI-5 are common among all *S. enterica* serovars (Table 1). To date, 23 SPI have been described although the functions of those genes contained within each island have not yet been completely elucidated (25, 26). SPI-1 and SPI-2 are of particular importance in *in vivo* infection (as shown in Table 1; Figure 2). The SPI encode effector proteins that are translocated directly into host cells across the plasma membrane type III secretion systems (T3SS-1 and T3SS-2) that provide *Salmonella* with the biochemical machinery to exploit this intracellular niche. T3SSs can also be used to secrete effector proteins into the surrounding environment to influence host cell physiology (27, 28) (Table 1).

**Table 1:** **Features and functions of SPI-1 through SPI-5 identified among all *S. enterica* serovars**.

Pathogenicity Island	Approximate size (kb)	Type secretion system	Features/functions	Reference
SPI-1	40	Type III secretion system (T3SS)	Invasion of intestinal epithelium; development of SCV; encodes effector proteins important for: actin cytoskeleton rearrangements; membrane ruffling; induce IL-8 and pathogen-elicited epithelial chemoattractant secretion	Ehrbar et al. (29), Hapfelmeier et al. (30); Ibarra et al. (31)
SPI-2	40	Type III secretion system (T3SS)	Survival within phagocytic cells such as macrophage; inhibits fusions between lysosomes and SCVs; endocytic trafficking inhibition; avoidance of NADPH oxidase-dependant killing by macrophages; encodes effector proteins: SpiC, SseF, SseG; encodes chaperone proteins: SscA, SscB, SseA; encodes translocon proteins SseB, SseC, and SseD	Waterman and Holden (32), Hapfelmeier et al. (30), Figueira et al. (33)
SPI-3	17		Intramacrophage survival; encodes macrophage survival protein MgtC; encodes Mg^2+^ transporter MgtB	Blanc-Potard and Solomon (34), Fierer and Guiney (16), Rychlik et al. (35)
SPI4	27	Type 1 secretion system (T1SS)	Mediates adhesion to epithelial cells; encodes genes *siiA-F* (*Salmonella* intestinal infection genes) and SiiE ~600 kDa non-fimbrial adhesion protein; role in oral virulence	Kiss et al. (36), Gerlach et al. (37), Rychlik et al. (35)
SPI-5	8		Encodes SopB (secreted by T3SS of SPI-1); encodes PipB (translocated by T3SS of SPI-2 to the SCV); important for *S*. Dublin virulence and induction of proinflammatory immune response in cattle	Zhang et al. (38), Rychlik et al. (35), Sabbagh et al. (25)

Salmonella pathogenicity islands-1 was originally thought to be important as an invasion-related cluster of genes required for oral virulence (39). More recently, additional functions have been described for this locus. SPI-1-induced activation of the host innate immune system results in inflammation and the recruitment of polymorphonuclear (PMN) cells across the intestinal epithelial barrier following the secretion of the effector protein SipA by *Salmonella*. The latter protein is required in conjunction with the cytokine, IL-8, and pathogen-elicited epithelial chemoattractant (PEEC) to recruit neutrophils as has been reported in cultured epithelial monolayers (40). The production of PEEC can be induced by SipA secretion or by direct addition of SipA to cultured intestinal epithelial monolayers leading to the recruitment of basolateral neutrophils to the apical epithelial membrane (41, 42). SPI-1 effector secretion also leads to NF-κB signaling- and caspase-1-mediated IL-1β/IL-18 activation (43). SipB, an SPI-1 encoded effector protein, which is translocated across the host cell membrane by T3SS-1, is critical for inflammatory disease *in vivo* (38) and is responsible for pyroptotic cell death, a rapid form of programed cell death associated with antimicrobial responses during inflammation that possesses both apoptotic and necrotic features (44, 45). SipB binds caspase-1 (IL-1β converting enzyme) in the cell cytosol resulting in the maturation of proinflammatory cytokines IL-1β and IL-18 into active peptides (46). Further studies have revealed that both caspase-1 and Ipaf deficient mice exhibit an increased susceptibility to typhoid fever, thereby demonstrating the protective proinflammatory role played by caspase-1 (47).

The proinflammatory activity of SPI-2 while less characterized has been shown to be important for intracellular persistence and systemic virulence in murine typhoid fever in addition to evading host phagosome oxidation mechanisms (48). T3SS-2 plays an important role in inflammatory disease, highlighting the involvement of SPI-2 in the onset of enterocolitis. SPI-2 functions by enabling the translocation of effectors across the membrane of the *Salmonella*-containing vacuole (SCV) in infected host cells. The genes encoding T3SS-2 are controlled by two-component regulatory systems such as OmpR–EnvZ and the SPI-2 encoded SsrA–SsrB. As many as 28 SPI-2 encoded effectors have been identified to date with many of these currently of unknown function such as SseK1-3 and SteA–B, D–E. SseF is involved in SCV localization and *Salmonella*-induced filament (Sif) formation. PipB2 is responsible for kinesin-1 recruitment to the SCV and Sif extension, whereas SspH2 and SteC are recruited to and involved in the formation of the SCV-associated F-actin meshwork, respectively (49). The Toll-like receptors (TLR) adapter, myeloid differentiation primary response gene (MyD88) is required for SPI-1 independent intestinal inflammation in mice (30).

## The Interaction of *Salmonella* with the Host

*Salmonella* invades both phagocytic and non-phagocytic cells including mononuclear phagocytic cells present in the lymphoid follicles, liver, and spleen. Epithelial cells and phagocytic cells such as dendritic cells, neutrophils, and macrophages identify specific pathogen-associated molecular pattern (PAMP) motifs and endogenous danger-associated molecular pattern molecules (DAMPs) present in the bacteria. Pattern-recognition receptors (PRRs), which include NOD-like receptors (NLRs) and TLRs, comprise the early components of the immune system that function to detect invading pathogens through PAMPs and DAMPs and signal to recruit and activate phagocytic cells such as neutrophils and macrophages (50, 51). These receptors trigger an immune response and are key to establishing an important network between the innate and adaptive immune systems. Bacterial DNA, flagella, and LPS are examples of PAMPs, which activate TLR4, TLR5, and TLR9 signaling in the host. LPS-induced TLR4 activation is important for triggering the inflammatory responses of the host. It also plays an important role in mounting an inflammatory response to intravenously administered LPS. Mice with mutations in TLR4-encoding genes exhibit an increased susceptibility to *Salmonella* infection irrespective of other *Salmonella* resistance *loci* (52, 53). Additionally, LPS plays an important role in the onset of sepsis during systemic infection as observed by its role in inducing inflammation in macrophages (54).

The immune system can be divided into two main parts: the innate or non-specific and the adaptive or specific components. The innate immune system is the first host challenge presented to invading pathogens whereas the adaptive immune system provides further protection in addition to an immunological memory, which enables a faster response upon repeat exposure to the same pathogen or antigen. In addition to cellular components such as phagocytic cells, there are humoral elements such as the complement system that make up the innate immune system. Additionally, anatomical features like the mammalian skin layer act as physical barriers to infection. The interplay between the innate and adaptive immune systems, including different types of cells and molecules such as cytokines and antibodies, form the totality of the host immunity.

Leukocytes of the innate immune system include phagocytic cells, namely dendritic cells, macrophages, and neutrophils, which can engulf foreign antigens, particles, or pathogens. These phagocytic cells are recruited following the release of specific cytokine signals. These cells serve an important role in the activation of the adaptive immunity, which usually assumes the presence of lymphocytes (55). Other cells, such as basophils, eosinophils, and mast cells are also part of the host innate immune system that contributes to the innate immunity.

During the initial stages of an inflammatory response, neutrophils and macrophages are recruited to the site of infection. Neutrophils phagocytose the invading pathogens and kill them intracellularly. Similarly, macrophages and newly recruited monocytes, which will differentiate into macrophages following signaling or chemical stimulation, also function by phagocytosing and killing the pathogens at the intracellular level. Furthermore, macrophages are capable of killing infected or self-target cells and can also induce further downstream immune responses through the presentation of surface antigens to signal and recruit other cells and cell types (56).

A common feature of salmonellosis is the notable inflammatory response elicited by the host innate immune system. Both the host and pathogen have evolved defense mechanisms that result in a complex cross-talk that culminates with the induction of the host immune response.

**Figure 3:**
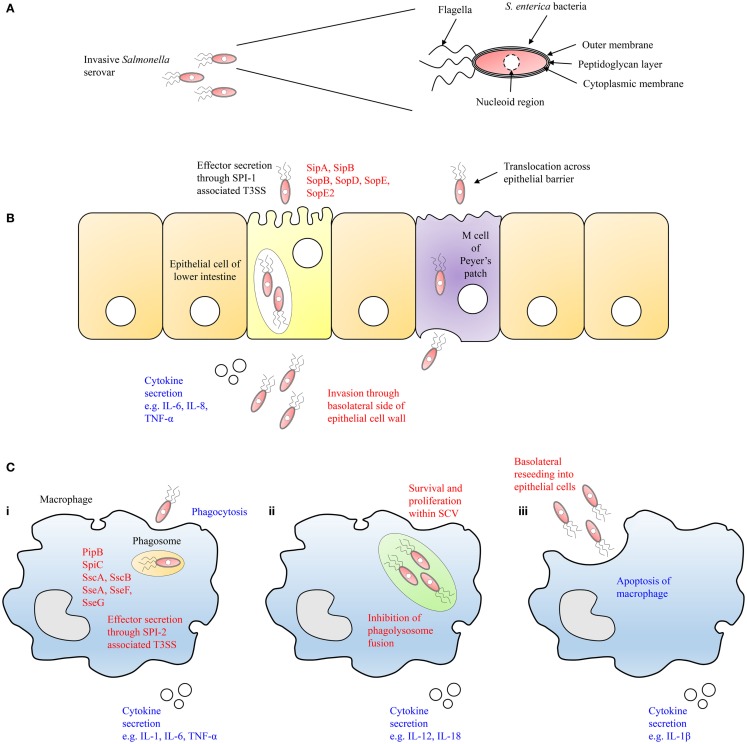
**Schematic illustration of the infection of epithelial cells of the lower intestine and macrophages by *Salmonella* is shown**. **(A)** The complex membrane structure of *Salmonella* allows it to survive until reaching the epithelial cell wall of the host in the lower intestine. **(B)** *Salmonella* then translocate across M cells of Peyer’s patches or actively invade epithelial cells by the secretion of effector proteins through the SPI-1 encoded T3SS-1. **(C) (i)** After crossing the epithelial barrier, *Salmonella* are engulfed by proximal macrophages that will secrete effector proteins into the cytosol of the cell via the SPI-2 encoded T3SS-2 and prevent fusion of the phagosome with the lysosome. **(ii)** Within the SCV, *Salmonella* will proliferate resulting in cytokine secretion by the macrophage. **(iii)** Finally, the macrophage will undergo apoptosis, and *Salmonella* will escape the cell to basolaterally reinvade epithelial cells or other phagocytic cells of the host innate immune system.

*Salmonella* species can cross the epithelial barrier by passive transport facilitated by dendritic cells, which extend pseudopods between local epithelial cells, or by active invasion. Upon reaching the lower intestine, the bacteria will adhere to the mucosal membranes and invade epithelial cells (57). One such site where this occurs is the microfold (M) cells of Peyer’s patches that are located in the small intestine where the bacteria will translocate across the epithelial barrier to the underlying follicles and mesenteric lymph nodes of the lymphoid tissue (58) (Figures 3A,B). During sustained bacteremia, secondary infections can occur due to the dissemination of the bacteria to other organs such as the gall bladder, liver, and spleen. The gall bladder serves as a reservoir in chronic cases of *S*. Typhi and *S*. Typhimurium infection (59, 60). Infection by invading bacteria can originate from both the blood and/or retrograde bile. Biofilm formation on gallstones is a reported avenue through which chronic carriage and shedding of *Salmonella* species can be established. These events set in motion a cycle of infection wherein bacteria basolaterally reinvade epithelial cells of the intestinal wall or are shed in feces. In time, the symptoms of salmonellosis will resolve. However, asymptomatic carriage of the bacteria can occur in patients for months or years with the potential to relapse in the future.

## Transmission of Infection

Following the ingestion of contaminated food, these bacteria will colonize the intestines by invading dendritic cells and enterocytes of the intestinal epithelium barrier. *Salmonella* species, which are successful in passing this barrier are confronted by proximal macrophages and may be phagocytosed, or actively invade the macrophages, using T3SS-1 and fimbriae, among other bacterial surface adhesins [H. (15)] (Figure 3Ci).

After being internalized by macrophages, *Salmonella* then reside within a membrane bound compartment distinct from the phagosome and lysosome known as the SCV. In this cellular compartment, *Salmonella* can survive and replicate in the absence of host antimicrobial defense mechanisms, thereby evading endosomal fusion with the NADPH oxidase complex (61) (Figure 3Cii). From within the SCVs, SPI-2 genes are expressed encoding T3SS-2, which enables *Salmonella* to translocate a range of effector proteins into the cytoplasm of the host cell including SigD/SopB, SipA, SipC, SodC-1, SopE2, and SptP leading to the rearrangement of the actin cytoskeleton. T3SS-2 has been described as necessary for systemic virulence in murine models and survival within macrophages (62). In contrast, systemic translocation of *S*. Dublin in cattle requires T3SS-1 but not T3SS-2 (63).

## Cytokine Responses and Signaling

Proinflammatory cytokines including the interleukins (IL-1β and IL-6), interferons (IFN-γ), and tumor necrosis factor (TNF-α) are synthesized and these act to promote systemic inflammation (64–67). IFN-γ, also known as macrophage activating factor (MAF), plays an important role in persistent infection as it influences the duration of macrophage activation. Secretion of IFN-γ is dependent on IL-18, also known as interferon gamma inducing factor, and is essential for establishing an early host resistance to infection with *Salmonella* (65, 68).

Macrophages are involved in both the innate and adaptive immune responses. Following exposure to specific cytokines, they undergo either classical (Th1) or alternative (Th2) activation. Classical activation by bacterial LPS or IFN-γ leads to alteration in the secretory profile of the cells through production of organic nitrate compounds such as nitric oxide (NO). Alternative activation by IL-4, IL-10, or IL-13 leads to the production of polyamines and proline inducing proliferation and collagen production, respectively. The presence of *Salmonella* within these cells leads to cytokine secretion and an inflammatory reaction or programed cell death through apoptosis (69, 70) (Figure 3Ciii).

Cytokine signaling, induced by the interaction of the host cells and bacteria, is crucial to the development and progression of salmonellosis. Cytokines are responsible for regulating both the innate and adaptive host immune responses. The equilibrium between pro- and anti-inflammatory cytokines controls the infection preventing damage to the host from prolonged inflammation. *In vitro* cell culture of bone marrow derived macrophages and primary cell lines have shown that *Salmonella* promotes chemokine and cytokine synthesis in both dendritic and epithelial cells as well as macrophages (69, 71, 72). Cytokines have a broad range of effects upon the host cell during infection. Chemokine C–C motif ligand (CCL2), IFN-γ, IL-12, IL-18, TNF-α, and transforming growth factor (TGF-β) confer protection during infection (73). Conversely, IL-4 and IL-10 interfere with the host defense mechanisms (74).

## Environment Adaptation

*Salmonella* adapt to the intracellular environment of phagocytic cells during infection. The transition from extracellular to intravacuolar environments involves global modulation of bacterial gene expression. The complete transcriptional landscape of intracellular *S*. Typhimurium following macrophage infection has been previously reported (75, 76). During replication in murine J774 macrophages, 919 of 4,451 *S*. Typhimurium genes are differentially expressed. Many of the *in vivo*-regulated genes are of unknown function suggesting novel macrophage-associated functions for intracellular growth (77).

It has been shown previously that *S*. Typhimurium requires glycolysis for infection of mice and macrophages and that glucose transport is required for replication within macrophages. During systemic infection of mice, *S*. Typhimurium replicates in macrophages within the SCV. Mutation of the *pfkAB-*encoded phosphofructokinase, the rate-limiting step in glycolysis, severely attenuates replication and survival within RAW 264.7 macrophages. Mutants with perturbed phosphoenolpyruvate:carbohydrate phosphotransferase systems or those unable to catabolize glucose exhibit reduced replication within RAW 264.7 macrophages (78).

*Salmonella* upregulates RpoS-dependent stress responses as well as other response mechanisms when challenged to grow in sublethal concentrations of the bile salt sodium deoxycholate (DOC). The latter is known to disrupt membranes, denature proteins, and damage DNA (79). It has been previously shown that *Salmonella* can pre-adapt to several stresses in order to survive the adverse conditions encountered, such as those encountered in a contaminated food matrix and any associated food production processes. Similarly, the subsequent ingestion of the bacterium by the host presents an array of challenges to the organism including acid, cold, osmotic, and peroxide stress (80).

## Pathological Symptoms

Prolonged activation of the innate immune system can have adverse effects, which include intravascular coagulation, systemic inflammation, and tissue injury. In severe cases, these symptoms can lead to death. An aggressive proinflammatory response to infection with *Salmonella* is not a common occurrence and it arises rarely in patients with typhoid fever. Unusual cases leading to intravascular coagulation do not present with readily recognizable clinical signs (81, 82). In these cases, the blood serum levels of IL-1β and TNF-α are lower when compared to that of patients infected with other Gram-negative bacteria (83).

Individuals suffering from typhoid fever exhibit a distinct peripheral blood metabolite profile, which has been elucidated by both microarray and transcriptional profiling techniques (66, 84). This profile diminishes following treatment and upon recovery the majority of individuals exhibit a peripheral blood profile similar to that of uninfected controls. Those who do not develop a typical peripheral blood profile following treatment may possess genetic mutations that render them incapable of mounting an appropriate immune response. These patients have been shown to be prone to relapse, reinfection, and in some cases become carriers (66).

## Immunodeficiency

There has been no evidence to support a correlation with susceptibility to typhoid fever and primary or acquired immunodeficiency. This is in contrast to infection with NTS serovars where infection causes high levels of morbidity and mortality in patients with primary or acquired immunodeficiencies such as HIV infection. It has been proposed that this difference is attributed to the manner in which signaling occurs via the PRRs. The production of IL-17 by T-helper 17 cells (Th17) among other cytokines (IL-21, IL-22, and IL-26) is important for the dissemination of NTS serovars but not *S*. Typhi (85, 86).

## Models of Infection

*S*. Typhi is a host-adapted pathogen, which infects humans causing typhoid fever. Investigating the interactions of this pathogen with the host has proved challenging as there are few animal models for typhoid fever that are of direct relevance to their human infection counterpart. This problem has been partially alleviated by the establishment of the murine *S*. Typhimurium infection model, which has been used to study typhoid fever. The immune responses and subsequent inflammation mounted by mice following an *S*. Typhimurium infection mimics those observed in human patients with typhoid fever as well as the subsequent intestinal pathology (87). Mice are inoculated orally or systemically by intravenous or intraperitoneal injection in addition to optional streptomycin pre-treatment (88). *S*. Typhimurium induced colitis in streptomycin-pre-treated mice is reminiscent of many symptoms of the human infection counterpart including epithelial ulceration and infiltration of PMN/CD18(+) cells (89). A comparison between streptomycin-pre-treated and untreated mice highlighted the drastic influence of streptomycin on resistance to colonization by *S*. Typhimurium whereby 100% of treated and none of the untreated mice excreted the bacterium in their feces (90). A disease with features reminiscent of typhoid fever can be observed in BALB/c or C_57_BL/6 mice when inoculated with *S*. Typhimurium due to a mutation in the *SLC11A1* gene, which encodes natural resistance-associated macrophage protein one (Nramp1). In contrast to this, chronic and persistent carrier states of infection can be studied using Nramp+/+ mice as they are resistant to infection with *S*. Typhimurium (25, 88). However, there has been no correlation identified in humans between *Nramp* alleles and susceptibility to typhoid fever as *S*. Typhimurium causes less severe disease symptoms in humans to that of *S*. Typhi. As a result, conclusions drawn from animal experiments must be interpreted carefully (91). Furthermore, it has been reported that *tlr11*^−^*^/^*^−^mice are more susceptible to infection by *S*. Typhimurium and can be infected with *S*. Typhi, which typically does not cause infection as TRL11 is normally expressed in mice but not in humans (92). Recently, an alternative *S*. Typhi murine model, which resembles human typhoid fever, was established using non-obese diabetic (NOD)-SCID IL2rγ^null^ mice, which have been humanized by engrafting human hematopoietic stem cells (hu–SRC–SCID mice). This model results in lethal infection with inflammatory and pathological responses, which mimic human typhoid fever (93).

As well as murine models of infection, the larvae of the wax moth *Galleria mellonella* (*G. mellonella*) have been used to study host–pathogen interactions with *Salmonella* species. Isogenic mutant strains of *S*. Typhimurium lacking known virulence determinants were tested to identify their role in pathogenicity. Interestingly, mutants depleted of either or both SPI encoded T3SS-1 and T3SS-2 exhibited no alterations in their virulence phenotype. Attenuation of the PhoPQ two-component signal transduction system resulted in reduced pathogenicity due to the lack of phoQ (94). As reported in murine models, mutations in the *hfq* gene, which encodes the chaperone protein Hfq that plays an important role in the binding of regulatory sRNA transcripts to their antisense targets attenuated the pathogenicity of *S*. Typhimurium in *G. mellonella*. Endoribonuclease RNase E and RNase III mutants show an attenuated virulence phenotype including impairment in motility and reduced proliferation inside *G. mellonella* (95).

Recently, zebrafish (*Danio rerio*) models have provided a unique opportunity to study the function of phagocytic cells such as neutrophils and macrophages. Transgenic zebrafish lines with fluorescently labeled leukocyte populations enable non-invasive imaging of the mechanisms by which different pathogens interact with macrophages and evade the host innate immunity (96). Similarly, 28 h old zebrafish embryos infected with DsRed labeled *S*. Typhimurium allowed for the precise location of the pathogen to be determined in a living host over a 3 day time course using multidimensional digital imaging microscopy. Lethal infection with *S*. Typhimurium residing and proliferating in both the endothelium layer of blood vessels and macrophages was observed (97).

## Future Perspectives

To date, there have been many studies elucidating the complex *Salmonella*–host interactome. Our understanding of the virulence determinants of *Salmonella* species and their mechanisms of action has been extended by the utilization of murine, *G. mellonella*, and zebrafish models of *S*. Typhimurium infection in addition to *ex vivo* cell culture methods. Despite this, further work is needed to determine the specific contribution of many of these regulators and virulence factors for which clear functions and roles have yet to be defined. Characterizing the pathogenesis of salmonellosis will be crucial to the development and implementation of future therapeutic strategies to treat this illness. The importance of which has been recently highlighted in reports on the emergence of antimicrobial resistance in *Salmonella* and many other bacterial pathogens (98).

## Conflict of Interest Statement

The authors declare that the research was conducted in the absence of any commercial or financial relationships that could be construed as a potential conflict of interest.
